# Disclosing the double mutualist role of birds on Galápagos

**DOI:** 10.1038/s41598-017-17592-8

**Published:** 2018-01-08

**Authors:** Jens M. Olesen, Christian F. Damgaard, Francisco Fuster, Ruben H. Heleno, Manuel Nogales, Beatriz Rumeu, Kristian Trøjelsgaard, Pablo Vargas, Anna Traveset

**Affiliations:** 1 Department of Bioscience, Aarhus University, Ny Munkegade 114, DK-8000 Aarhus, Denmark; 2 Institut Mediterrani d’Estudis Avançats (CSIC-UIB), Esporles, Balearic Islands, Spain; 3 Center for Functional Ecology, Department of Life Sciences, University of Coimbra, Coimbra, Portugal; 4 Instituto de Productos Naturales y Agrobiología (CSIC-IPNA), La Laguna, Canary Islands, Spain; 5 Department of Chemistry and Bioscience, Aalborg University, Aalborg, Denmark; 6 Real Jardín Botánico (CSIC-RJB), Madrid, Spain

## Abstract

Life on oceanic islands deviate in many ways from that on the mainland. Their biodiversity is relatively poor and some groups are well-represented, others not, especially not insects. A scarcity of insects forces birds to explore alternative food, such as nectar and fruit. In this way, island birds may pollinate and disperse seed to an extent unseen on any mainland; they may even first consume floral resources of a plant species and then later harvest the fruit of the same species. Through this biotic reuse, they may act as double mutualists. The latter have never been studied at the level of the network, because they are traditionally considered rare. We sampled pollination and seed-dispersal interactions on Galápagos and constructed a plant-bird mutualism network of 108 plant (12% being double mutualists) and 21 bird species (48% being double mutualists), and their 479 interactions, being either single (95%) or double mutualisms (5%). Double mutualists constitute the core in the pollination-dispersal network, coupling the two link types together. They may also initiate positive feedbacks (more pollination leading to more dispersal), which theoretically are known to be unstable. Thus, double mutualisms may be a necessary, but risky prerequisite to the survival of island biodiversity.

## Introduction

The poor presence and small population sizes of many groups of species on oceanic islands compared to mainland standards may jeopardise survival of island animals and push them out into a wider food niche, by including more kinds of suboptimal food into their diet^1–4^. They do so by either consuming more species or even consuming more kinds of food items of the same species, *e*.*g*. herbivores may consume not just foliage of a plant but also its fruit.

In particular, insects serve as a plentiful source of food to many animals, *e*.*g*. more than half of all bird species in the world have insects as a favourite on their menu roster^5^. This, however, turns into a problem on oceanic islands, which often have a low density of insects^6–8^, but a high abundance of some vertebrate insect-consumers, especially density-compensating birds and lizards^9^. In mainland systems, birds and lizards are assumed to be either strict insect eaters, nectar drinkers or fruit/seed eaters, whereas on islands they turn more omnivorous, consuming both floral resources, fruit and seed, as well as insects^10–12^. This is termed interaction release^4^, and is a kind of ecological release, involving an increase in the diversity of biotic interactions established by species. When small vertebrates exhibit interaction release, they may become pollinators and seed dispersers to many plants, which also suffer from this paucity of insects for their pollination. In a world of such scarcity, island vertebrate consumers may even “reuse” the same food plants, that is, first harvest their floral resources and then later in the season the fruit of these and thus serve as both pollinators and seed dispersers of the same plant species, *i*.*e*. they become ‘double mutualists’ (*sensu* ^13,14^). Interaction release and also omnivory in general may affect ecosystem stability^15,16^, but how this is modified by double mutualisms is unknown. Double mutualisms are poorly studied, but have been regarded as anecdotal and only single cases in a few ecosystems are known^14,17,18^. Here, however, we disclose a frequent double pollination/dispersal mutualist role played by many members of the land bird community on the Galápagos Islands; we report the extent of this phenomenon on the archipelago, and briefly discuss its consequences for island networks and their stability in general. By stability we here mean low variance in network parameters. Finally, in a broader network context, we identify double mutualists as a special kind of network connector nodes, lowering the overall heterogeneity of nature.

## Results

We imported all observed pollination and seed dispersal interactions into a total plant-bird mutualism matrix, representing the year-round interactions of the whole archipelago (Supplementary Dataset S1). This matrix included 108 plant species (only taxa identified to species were included; 33% are human introductions) and 21 bird species (only Smooth-billed Ani (*Crotophaga ani*) and Cattle Egret (*Bubulcus ibis*) are introduced). The matrix had a total of 479 links (Table 1; each double mutualism is scored as a single interaction). Thus connectance of the total Galápagos plant-bird mutualism matrix became 479 × 100/(108 × 21) = 21%.

**Table 1:** Number and percentage of species and links in the total plant-bird mutualism matrix and its different functional combinations, Galápagos. p: pollination link; s: seed-dispersal link; ps: double mutualism.

**Numbers**	2 pollinators	**10 double mutualists**	7 pollinators-dispersers	2 dispersers
61 pollinated plants	3p	185p	58p	
**13 double mutualistic plants**	2p	42p + 25 ps* + 14s	24p + 9s	4s
7 pollinated-dispersed plants		21p + 8s	12p + 1s	1s
27 dispersed plants		52s	12s	6s
**Percentages**	**9.5% pollinators**	**47.6% double mutualists**	**33.3% pollinators-dispersers**	**9.5% dispersers**
56.5% pollinated plants	0.63%p	38.62%p	12.11%p	
**12.0% double mutualistic plants**	0.42%p	**8.77%p + 5.22%ps + 2.92%s**	5.01%p + 1.88%s	0.84%s
6.5% pollinated-dispersed plants		4.38%p + 1.67%s	2.51%p + 0.2111%s	0.21%s
25.0% dispersed plants		10.86%s	2.51%s	1.25%s

*Each double mutualism is counted as one interaction.

10 birds and 13 plants were double mutualists.

The most generalized double mutualists were Small Ground-finch (*Geospiza fuliginosa*) and Medium Ground-finch (*G*. *fortis*), involved in six and five double mutualisms, respectively.

All species were sorted into four groups: Single pollination mutualists (61 plant and two bird species), single dispersal mutualists (27 plants and two birds), pollination-dispersal mutualists (seven plants and seven birds; *i*.*e*. species with both pollination and seed dispersal links, but these were directed to different species) and double mutualists (13 plants and ten birds, *i*.*e*. species with some of their pollination and dispersal links directed to the same species). Thus, a substantial proportion (18%) of all plant and bird species were double mutualists, especially among the birds (48%).

**Figure 1:**
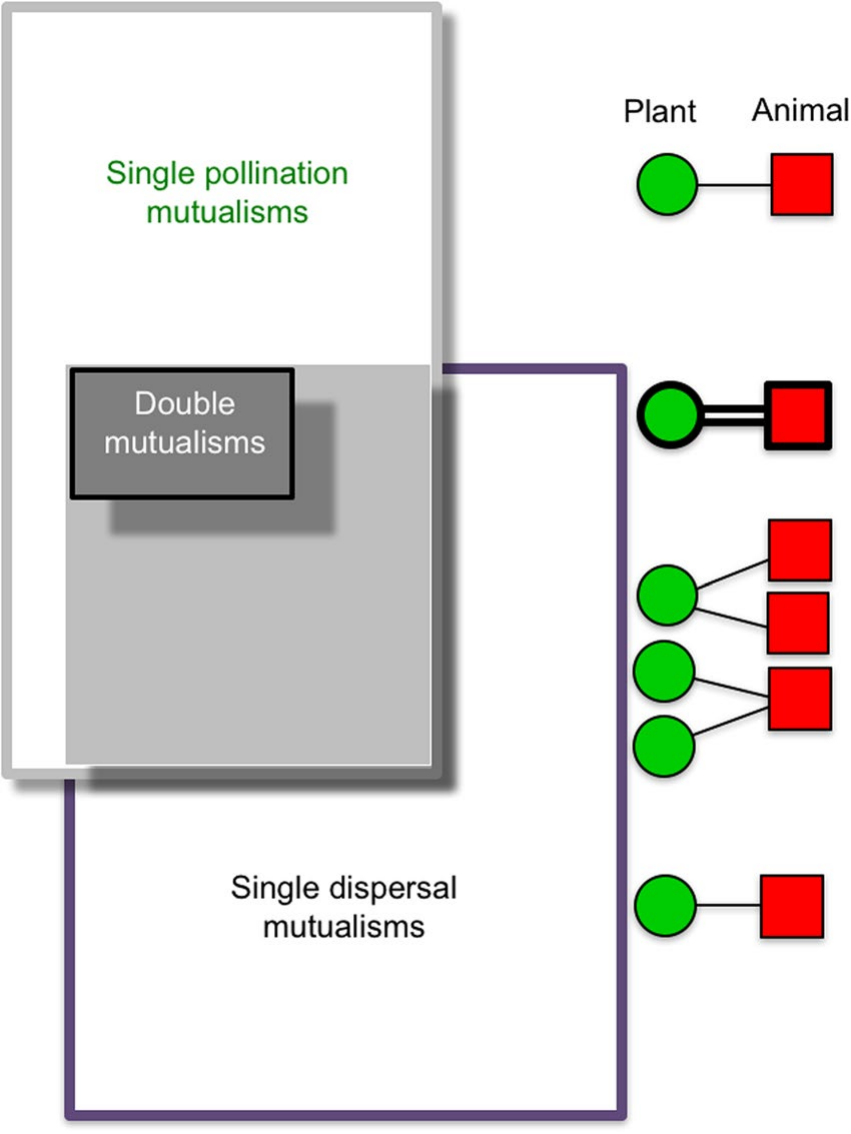
Plant-bird mutualism matrix, Galápagos. This network is a composite of a pollination (grey frame) and a seed-dispersal matrix (violet frame). The overlap area (grey-coloured) of the two matrices includes all species involved in both pollination and dispersal. The dark grey area within the overlap is the ‘double mutualism submatrix’, which includes all double mutualists and their links. A double mutualism is defined as a combined pollination and seed dispersal interaction between the same two species (‘green plant’ and ‘red bird’ in right column). In addition, double mutualist species may have single pollination and seed dispersal links to other species. Species interacting in the light grey area outside the double mutualism submatrix have both pollination and seed dispersal links, but not to the same species. The different link categories are illustrated in the column on the right.

All 479 links were sorted into three kinds: Single pollination mutualisms (73%), single dispersal mutualisms (22%), and double mutualisms (5%) (Table 1, Fig. 1, Supplementary Dataset S1).

The pollination and dispersal matrices had an overlap area; the grey area in Fig. 1. Here all species were involved in both pollination and seed dispersal, but not always to the same species. The overlap area included 20 plant species and 17 bird species and their 156 links, which were either single pollination links, single dispersal links or double mutualisms (Table 1). Thus, connectance of the overlap area became 46%.

**Figure 2:**
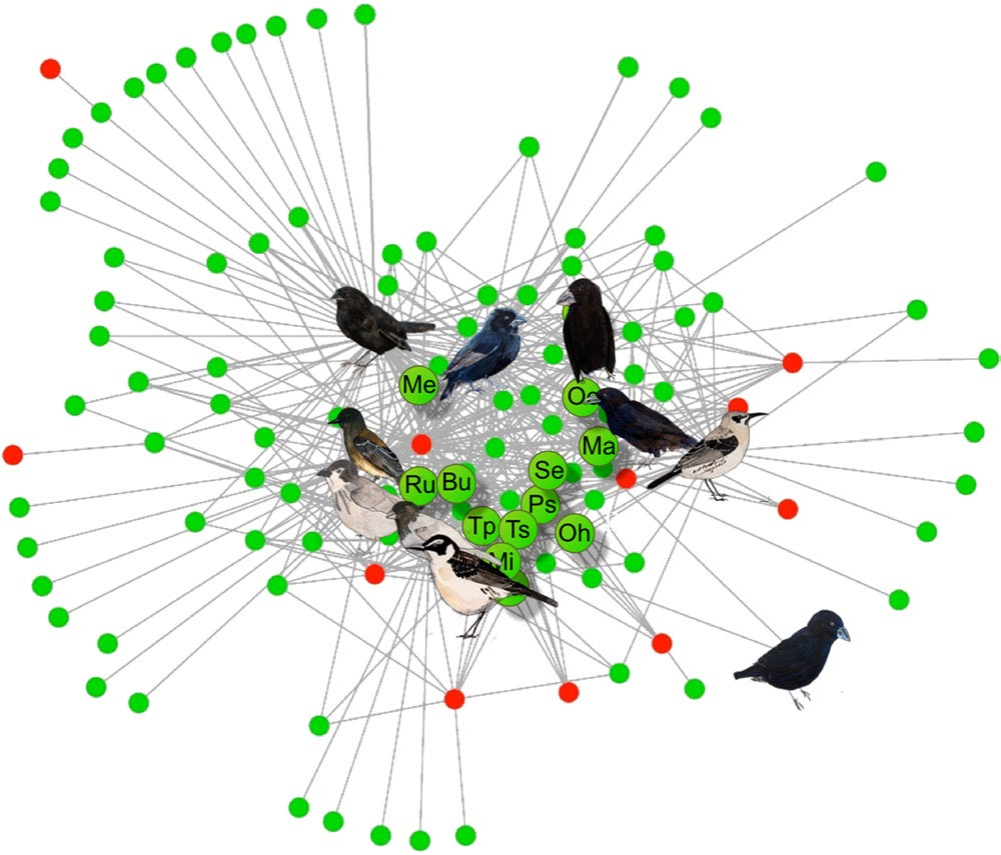
Plant-bird mutualism network, Galápagos. Double mutualistic plant nodes are enlarged and coloured green, their letter coding is explained in Supplementary Dataset S1. Birds are from upper left and clockwise: Small Ground-finch (*Geospiza fuliginosa*), Medium Ground-finch (*G*. *fortis*), Large Ground-finch (*G*. *magnirostris*), Common Cactus-finch (*G*. *scandens*), San Cristóbal Mockingbird (*Nesomimus melanotis*), Galápagos Mockingbird (*N*. *parvulus*), Small Tree-finch (*Camarhynchus parvulus*), Woodpecker Finch (*C*. *pallidus*), and Galápagos Flycatcher (*Myiarchus magnirostris*), and in the lower right corner Large Cactus-finch (*G*. *conirostris*) (Ill. Jens M. Olesen).

Inside the overlap area, we had the smaller double mutualism submatrix; the dark-grey area in Fig. 1. Here, all species were involved in at least one double mutualism. It included 13 plant species and ten bird species and their 25 double mutualism links (Table 1). All 25 links were true double mutualisms between the same plant and animal species, and both interactions in each double mutualism were observed on the same island. In addition, the double mutualism submatrix included 42 single pollination links and 14 single dispersal links, *i*.*e*. a double mutualism submatrix connectance of 62%. Thus, by moving from the total matrix, to the overlap area and further into the double mutualism submatrix, connectance tripled from 21%, to 46% and to 62%, *i*.*e*. the double mutualisms and their bird and plant nodes constituted the most link-dense and thus most coherent part of the total network (Fig. 2).

We examined if there was any positive or negative association between pollination and dispersal, *i*.*e*. assuming a bird was both a pollinator and a disperser, how would that affect its chance of being a double mutualist as well, and *vice versa* for a plant? We ran 50,000 randomizations of the spread of links in the overlap matrix (Fig. 1) and counted the number of double mutualisms in both the overlap area and in the smaller double mutualism submatrix (Fig. 1). If pollination and dispersal links were established independently of each other, the probability of achieving the actual 25 double mutualisms would be 0.13 in the overlap area and 0.04 in the double mutualism submatrix (Supplementary Table S1). Thus, if a bird species was involved in both kinds of interaction it was slightly more likely to be to the same plant species, and the same for plants, than if interactions were randomly distributed, *i*.*e*. pollination and dispersal were weakly coupled ecosystem functions and thus, both functions might have the same underlying drivers, *e*.*g*. abundance.

Data on bird abundance, their body (*i*.*e*. beak and forehead) pollen load and the seed load in their droppings are given in Supplementary Table S4 and data on bird diet, body size, geographic range etc. in Supplementary Dataset S2. According to the literature^5^, 14 Galápagos bird species are regarded as omnivores, four as arthropod-eaters, two as herbivores, and one as carnivore (Supplementary Table S2). However, our study demonstrated a general move of the bird fauna towards floral resources and fruit consumption. The four species, which we did not score as being both pollinators and dispersers, were the omnivorous Smooth-billed Ani (*Crotophaga ani)*, Sharp-beaked Ground-finch (*Geospiza difficilis*) and Española Mockingbird (*Nesomimus macdonaldi*), and the carnivorous Cattle Egret (*Bubulcus ibis*). Smooth-billed Ani, Española Mockingbird and Cattle Egret were the largest of all 21 birds, and Española Mockingbird was only observed on one island.

Abundance *A*_*B*_ of birds (using ringing data as a proxy for abundance) was strongly positively correlated with their archipelago linkage level *L*_*B*_ (Supplementary Dataset S1 and Supplementary Table S4; 19 species, *L*_*B*_ = 9.67 + 4.27 *A*_*B*_ – 0.07 *A*_*B*_^2^, *R* ^2^ = 0.81, *F* = 40.4, *P* < 0.001), *i*.*e*. the more abundant birds made more interactions to plants. *A*_*B*_ was 2.9 times higher for double mutualists than for single mutualists. However, the difference was not significant (Wilcoxon Rank sum test *W* = 23.5, *P* = 0.09), but there was a trend for a higher double mutualist bird abundance. Abundance *A*_*P*_ of plants (using summed 2010–2011 counts of flowers or flower heads from Fernandina, Pinta, Santiago, Santa Cruz and San Cristóbal as a proxy for abundance; data are not included in supplementary material) was not correlated with their archipelago linkage level *L*_*P*_ (45 species, *L*_*P*_ = 2.08 + 0.44 ln *A*_*P*_, *R* ^2^ = 0.05, *F* = 3.46, *P* = 0.070). However, *A*_*P*_ was 6.6 times higher for double mutualists than for single mutualists (Wilcoxon Rank sum test *W* = 191, *P* = 0.049; note the *P*-value). Thus, abundant bird species explored a higher diversity of plant species, but not necessarily both floral and fruit resources. Abundant plants, on the other hand, did only weakly, and not statistically significantly, respond to bird diversity by making more interactions.

We used quantitative data, *i*.*e*. body load size of pollen and number of seeds in bird droppings, to compare species dependence and strength of single and double mutualists/mutualisms (Supplementary Tables S4 and S5). We did that for the pollination and seed dispersal networks separately, because we could not find any adequate “exchange rate” between the two link currencies, *i*.*e*. pollen and seed loads, that would allow us to merge the two kinds of network (see Materials and Methods). Only species strength differed significantly between single and double mutualists. For both network types, double mutualists had the highest species strength, which means that their interaction partners depended more on them than on single mutualists.

We investigated if the network was nested, *i*.*e*. if each species more often interacted with a subset of those species, which more generalized species interacted^19^. The total network was significantly nested (*NODF* = 31.2, *P* < 0.01), and all double mutualisms except one were located in the link-dense core of the network, *i*.*e*. the interaction core between the most generalized species (Supplementary Dataset S3). Only one double mutualism was between relatively specialized species, namely Heller’s Prickly Pear Cactus–Large Cactus Finch (*Opuntia helleri*–*Geospiza conirostris*). The high centrality (measured as high linkage level *L*) of the double mutualists observed in the nestedness analysis was confirmed by their higher network betweenness centrality score (double mutualists: 0.0481) compared to the other species of the network (single mutualists: 0.0028, and pollinator-disperser mutualists: 0.0148) (Supplementary Fig. S1), which means that the double mutualists played a strong role, gluing the two kinds of network together.

Finally, we also investigated if the network was modular, *i*.*e*. if the network consisted of distinct and highly linked species groups or modules^20^. However, the total network was not significantly modular ((*M* (observed)* = *0.2535; mean *M* (random) ± standard deviation of 100 random runs = 0.2598 ± 0.0051, *P = *0.11), *i*.*e*. the network functioned as one single coherent module.

## Discussion

### The Galápagos bird-plant mutualism network

In the Galápagos plant-bird mutualistic network, double mutualists constitute a substantial proportion of all plant and bird species (18%), especially the birds (48% of the bird community)^21,22^, which irrespective of their traditionally assigned feeding preferences move towards double mutualism. The double mutualists and their interactions dominate the link-dense core of the mutualism network (high connectance, significant nestedness) and as a result, the network operates as one single, coherent module (no modularity). The high species strength of double mutualists also tells that their interaction partners (the single mutualists) depend on them both for their pollination and seed dispersal, and that these two ecosystem functions are coupled. One of the drivers of this may be high species abundance and thus high intraspecific competition for preferred food and pollinators. The high betweenness of double mutualists also suggests a coupling of the two ecosystem functions, again underlining that the two kinds of network are tightly glued together and operate as one functional unit.

### Galápagos vertebrates as double mutualists

In Galápagos, 17 out of 21 bird species pollinate and disperse the seeds of plants, and ten of the birds are even involved in at least one double mutualism with some of the 13 double mutualistic plant species. Smooth-billed Ani is one of the four birds, which was not observed to harvest floral rewards. However, it does so on the island of Cuba, where it has been observed to visit flowers^J. M. Olesen *unpubl*^. Our Galápagos results are an example of biotic reuse or interaction release^4^, which confirms that island animals widen their food niche both by including more food species, but also by using more kinds of food from the same plant species; and, *vice versa*, plants also reuse the same animal species for both pollination and dispersal^11^. We argue that the commonness of double mutualisms at the network level perhaps is a simple consequence of resource scarcity and low biodiversity on islands, especially among the most abundant species suffering the most from intraspecific competition. Even more Galápagos bird–flower/fruit interactions are known from the literature^10,23^.

Besides the birds, other Galápagos animals may also be part of this concoction of plant mutualists, *e*.*g*. the lava lizards (*Microlophus* spp.), the iguanas, the giant tortoises, and various mammals. The lizards are both pollinators and seed dispersers^24^, and the last three groups are important dispersers^25,26^. Even the Galápagos snakes may disperse seeds, either directly consuming fruits, *e*.*g*. snakes consume the pulp of the invasive squash *Momordica charantia* on the Galápagos island Santa Cruz^D. Meribeth *pers*. *com*.^ or perhaps indirectly, as shown in Africa for snakes, predating seed dispersers^27^. The endemic rodents (Oryzomyini: *Nesoryzomys* spp.) may also visit or consume flowers and fruit^28^. In fact, in Costa Rica a species of a related genus *Oryzomys* is a pollinator^29^. Finally, Galápagos have two bat species (family Vespertilionidae: *Lasiurus* spp.) supposed to be insect-eaters. We inspected eight specimens of one of them, *L*. *cinereus*, in the collection at the Charles Darwin Station, and all had pollen on their forehead^*unpubl*.^. This is a surprising finding, because hitherto in other parts of the world, flower visitation in Microchiropteran bats is only known from the New Zealand endemic *Mystacina tuberculata*, the Neotropical Phyllostomidae, and two species in Vespertilionidae (*Platyrrhinus lineatus* and *Antrozous pallidus*)^30–32^, of which at least *Mystacina* and Phyllostomidae possess morphological adaptations for nectar foraging. Thus, like many birds and lizards on insect-poor islands^9^, bats may turn to additional or alternative food sources, *e*.*g*. plant material, in order to survive and, as we discovered, this also seems to be the case on Galápagos.

The observation of reuse of island biotic resources may be taken a step further. For example, the Small Tree-finch (*Camarhynchus parvulus*) and the Galápagos Flycatcher (*Myiarchus magnirostris*) are generally regarded as arthropod-eaters or omnivores, but are here scored as double mutualists. However, they may even be triple mutualists, using the same plant species as a source for floral rewards, seed/fruit and herbivorous insects, *e*.*g*. the flycatcher consumes floral resources and seed/mesocarp from the tree *Bursera graveolens*, but we also observed it gleaning the canopy of the tree for insects, of which some may be herbivores and hence, the bird may benefit the tree in three ways. Another example of a triple mutualism is the relationship between the Large Cactus-finch and *Opuntia* spp.^23^. However, triple mutualisms may also include antagonistic foraging, such as bark stripping by birds^23^. We believe triple mutualisms to be common on islands and future studies should look for triple mutualisms on both islands and mainland. These preliminary observations suggest that the trophic levels of small island networks are very entangled and blurred.

We presume double mutualisms to be prevalent on remote islands worldwide, and the trophic downgrading of arthropod-eaters to omnivory may be a simple response to a shortage of animal food^33^ (however, see^23^, p. 212). Downgrading also reduces the minimum required home range size, which further may boost population size and thus resistance against extinction. However, further studies are required to investigate double mutualism on Galápagos and other remote islands.

### Global distribution of double mutualisms

The global distribution of double mutualisms is described in Fuster *et al*. (*submitted*), which lists more than 300 cases, especially from the tropics. They occur mostly in insect-poor environments, *e*.*g*. high mountains and oceanic islands. The latter have several times more double mutualisms than mainland sites. Globally, the double mutualists are a few ants and bees^17,34^, *c*. 50 lizard species^14,A. Valido & J. M. Olesen *unpubl*.^, 14–51 flying foxes^18,35^, an unknown number of Phyllostomid bats and finally, birds from many families^36,37^. Thus, for sure, our island double mutualism observations can be repeated on other remote islands, but whether it also is a widespread community and network phenomenon is unknown. Before the introduction of the Brown Snake to the Island of Guam, this island had a rich fauna of birds, lizards and flying foxes^38,39^, and so had Mauritius^40^, Polynesia^41^ and New Zealand^11^ and some of them may, although this is speculative, have served as double plant mutualists. As an additional network example, we compiled data from a subset of the rich literature about New Zealand bird-plant mutualisms and made a preliminary interaction matrix of 95 plant species, 31 bird species and 400 links (Supplementary Table S3). The proportion of double mutualisms was 9.8%, *i*.*e*. almost twice as high as the 5.2% from Galápagos, supporting our conclusion about double mutualisms being an island network phenomenon. However, until we get similar mainland data, we cannot say if Galápagos and New Zealand are exceptions or the rule.

Endemic island plant mutualists such as birds, lizards and tortoises are exceedingly vulnerable to introduced species^42^, and their extinction has serious consequences for the reproduction of native plants^43^, *i*.*e*. invaders may destroy the double mutualism core of the island plant-vertebrate mutualism network. Although the loss of endemic vertebrate mutualists may be mitigated by introduced analogue species replacing the loss and thus tinkering the missing ecosystem functions^44,45^.

### Stability of island mutualism networks

**Figure 3:**
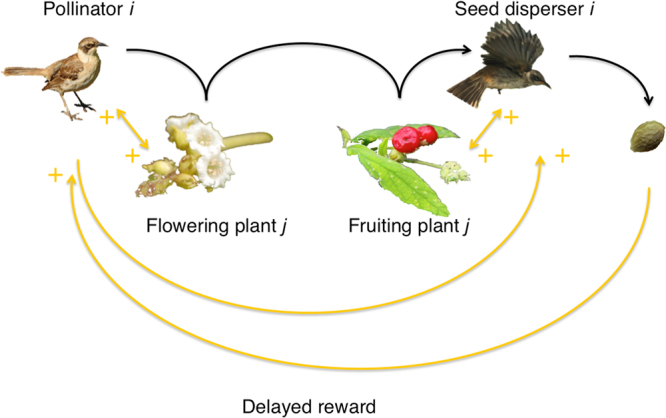
General plant-vertebrate double mutualism motif. The temporal delay within the motif is expected to vary with species (Ill. Ruben Heleno).

**Figure 4:**
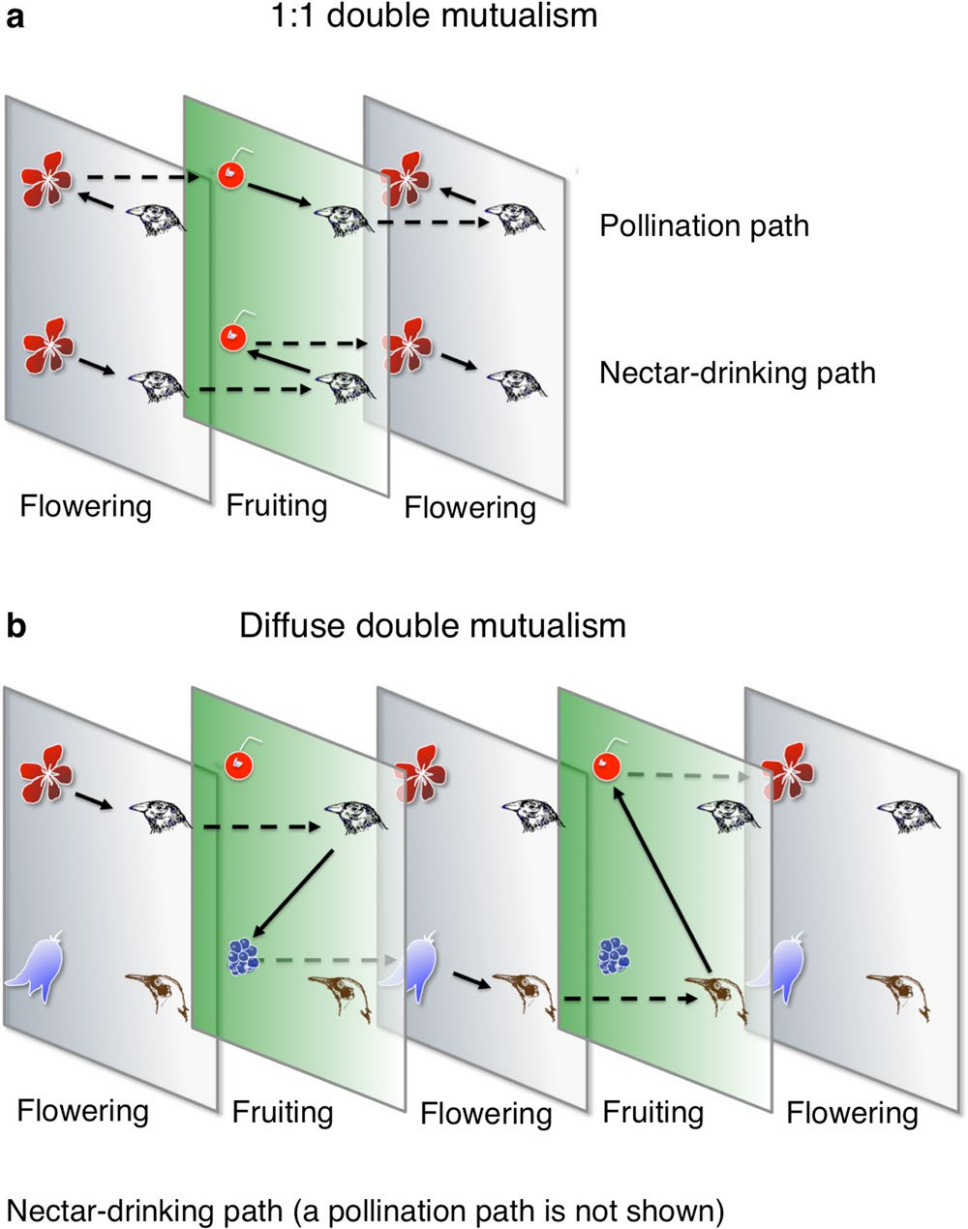
General plant-bird double mutualisms through alternating flowering and fruiting seasons. A solid arrow indicates a plant-bird interaction, and a dashed arrow indicates a within-species positive effect in the subsequent season. (**a**) A 1:1 double mutualism involving two interacting species. The double mutualistic interaction is shown as two opposite-running paths (pollination and nectar-drinking). (**b**) A diffuse double mutualism involving more than two species. Here for clarity reasons, only the nectar-drinking path is shown (Ill. Jens M. Olesen).

Double mutualisms couple up pollination and seed dispersal of a plant species together in a positive feedback loop (Figs 3, 4), and such a loop may be seen as a force of divergence, amplifying any initial disturbance^46^. Networks with loops or recurrent networks may be unfolded in time (Fig. 4), making it possible to track the paths of effects from species to species. If an animal makes more floral visits to a plant species, the latter may achieve more pollination and produce more fruit, which then may attract more harvesting animals and ultimately more seeds get dispersed, that is the pollination path in Fig. 4a. This may be a likely outcome, because (1) the association between pollination and dispersal in the double mutualism submatrix is positive, and (2) a substantial portion of the birds and plants are involved in double mutualisms. Consequently, the two processes pollination/nectar-drinking and seed dispersal/frugivory-granivory will enforce each other. At least in theory, this is a likely scenario. In the bird part of the loop, we also see a positive feedback: if a plant produces more floral rewards, a visiting animal harvests more energy, may produce a larger clutch, which harvests more fruit, and the animal achieves a higher reproductive success, that is the nectar-drinking path in Fig. 4a. In general, such positive feedback loops are regarded as unstable^46^. Most Galápagos animal mutualists pollinate some plant species and disperse the seeds of others (the red-coloured species in Supplementary Dataset S1), and in those cases, weaker and more complex, multi-species positive loops may appear, expected to be weaker because the strength of a feedback loop is the product of all its individual interaction strengths^46^; one example is shown in Fig. 4b. On the other hand, if just one link drops out, the feedback loop may break apart. If the double mutualist also is an herbivore, *e*.*g*. the Vegetarian Finch (*Platyspiza crassirostris*) (Supplementary Fig. S2a), the sign of the entire positive feedback loop switches, turning it into a negative feedback, that now may act as a stabilizer against disturbance. Thus, this interaction release may have severe consequences for stability, *i*.*e*. on the population growth rates of all species involved in the network.

The same goes for interaction cascades, which we define as being broader than trophic cascades^47^, because they include non-trophic interactions such as pollination. If predators prey on pollinators and seed dispersers they have a negative indirect effect upon the plants, whereas if their prey are herbivores their indirect effect may become positive^48,49^ (Supplementary Fig. S2b).

Merging different mutualistic networks, as done in our link analysis, may also reveal unforeseen consequences to total network stability. Theoretically, it is predicted that if species linkage levels in each of the two networks are positively correlated (*i*.*e*. if, for example, a pollination generalist also tends to be a dispersal generalist), then perturbations should propagate easily throughout the total network, whereas if linkage levels are negatively correlated this should not happen^48^. In a correlation analysis of linkage levels for the species being involved in both pollination and seed dispersal in the Galápagos networks (Supplementary Dataset S1), we find that bird linkage levels in the pollination and the seed-dispersal network were positively correlated (*F*_1,15_ = 16.5, *P* < 0.01), whereas plant linkage levels were uncorrelated (*F*_1,21_ = 1.21, *P* = 0.29). Thus, perturbations between the two kinds of network may propagate through the bird community and less so through the plant community.

How all these stability considerations influence the final bottom line for the overall network effects of double mutualisms, including the direct and indirect links between predators and plants, we do not know. However, we do conclude that island networks may be fragile because of a dominance of double mutualists in the network core (*i*.*e*. biotic reuse being common), positive feed back loops, and strong omnivory, but also that we are far from a deeper understanding of their dynamics.

### Connectors at different scale level in a hierarchical, modular network

The plant-bird mutualism network of Galápagos is organized into one single coherent module. If other groups of plant-consuming vertebrates are included, significant modularity may appear^50^. However, double mutualists with their high betweenness values may counteract this, because of their functional duality (pollination and dispersal), allowing them to connect different network parts together.

**Figure 5:**
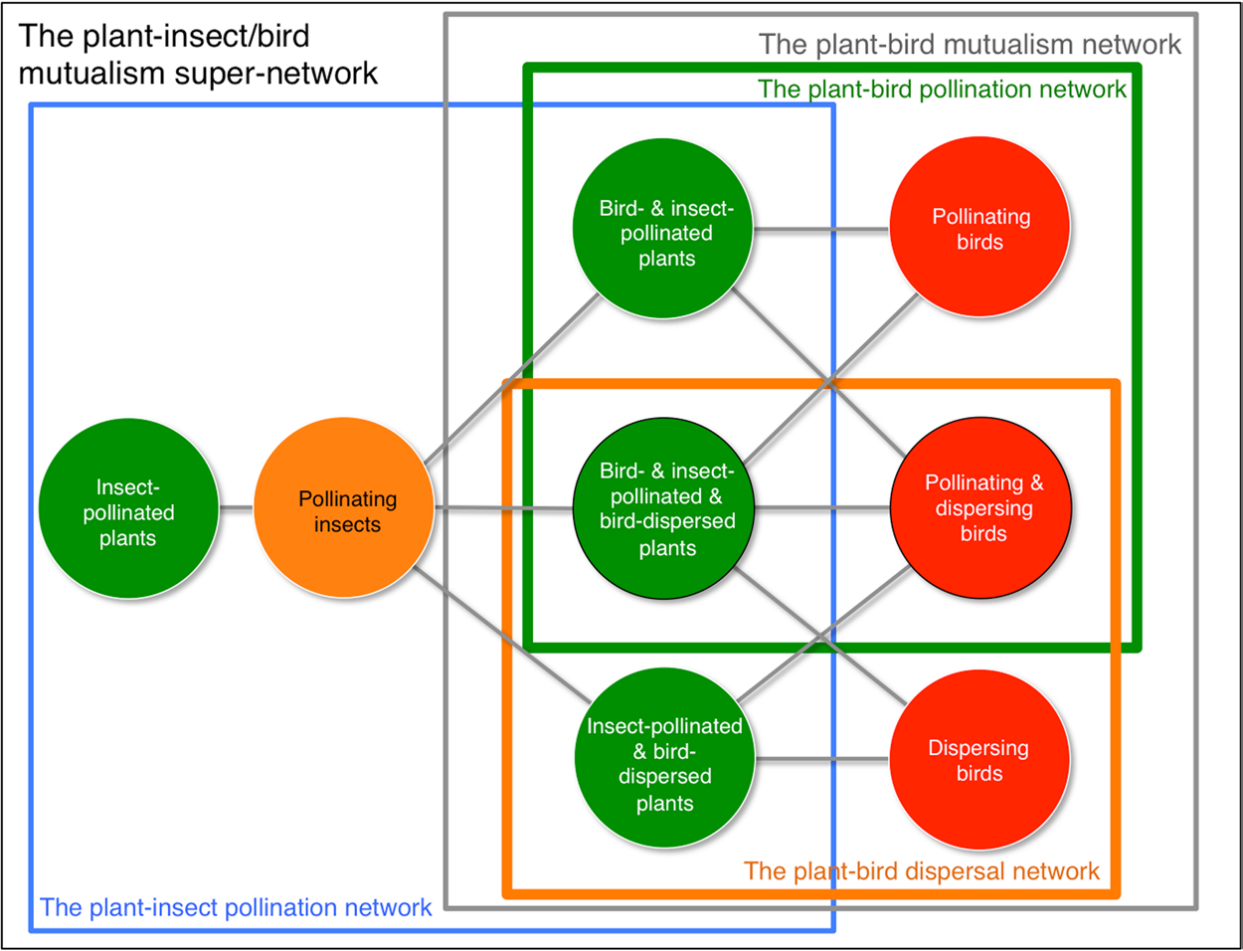
Hierarchical structure of the plant-insect/bird mutualism super-network, Galápagos. This network consists of a plant-insect pollination network (blue frame) and a plant-bird mutualism network (grey frame), and the latter is further composed of a plant-bird pollination network (green frame) and a plant-bird dispersal network (orange frame). Green, orange and red circles represent communities of plants, insects and birds, respectively. Double mutualists are located within the overlap between the green and orange frames.

In Galápagos, the plant-bird mutualism network is a part of a larger terrestrial super-network including different kinds of biotic link (Fig. 5); and with its coupling to sea and vagrant birds and mammals, this super-network becomes linked to regions outside the Galápagos archipelago, *e*.*g*. several seabirds migrate between North and South America and Galápagos^5^. These seabirds bring organic matter to the islands, sustaining a coastal decomposer network of invertebrates, which, in turn, are consumed by some of the landbirds in our network, *e*.*g*. *Geospiza* species may forage along the seashore^*pers*. *obs*^. Thus, our island study opens a window, albeit small, out towards a broader understanding of the interaction structure of nature on a global scale.

An initial step towards constructing a Galápagos super-network may be to merge our network with the Galápagos plant-insect pollination network^21^ (Fig. 5). At present, this larger plant-animal mutualism network encompasses 1,354 interactions between 162 plants and 233 animals of which 212 are insects. It consists of six modules; one is mainly our bird pollination/dispersal module, and the remaining five are mainly insect pollination modules^*unpubl*.^. In this network, 42 species are connectors (16 plants, 20 insects and six birds), linking modules together. The plant connectors link to both insects and birds, because of the way the network is constructed.

Thus, at each hierarchical level (*i*.*e*. lowermost level–bird pollination or dispersal; intermediate level–general vertebrate (birds, lizards, tortoises etc.) mutualisms; and uppermost level–animal (vertebrates and insects) mutualisms), the Galápagos ecological networks are built from modules linked together by connecting species. The latter have a richer link diversity and functional versatility than other species, and because of that they may operate on several hierarchical levels, gluing species and modules of different functionality together^51,52^, ultimately increasing overall network interdependency. That means connectors reduce the level of modularity and thus overall heterogeneity in their networks. One strong group of connectors is the double mutualists, and their high prevalence, at least on Galápagos and New Zealand, is a signature of insularity (Fuster *et al*. *submitted*), being characterized by resource limitation, interaction release and biotic reuse.

### General conclusions

We see a scenario, where a paucity of insect food and perhaps also large predators force or allow birds and other vertebrates to widen their food niche and act as connectors or even as double mutualists, thereby reducing network heterogeneity, which ultimately may destabilize the network^53^. The strong occurrence of species on islands being double mutualists is an unprecedented phenomenon in island biology (Fuster *et al*. *submitted*). However, it is a finding affecting not only our view of island ecosystems, but also our understanding of the importance of multi-functionality in ecosystems in general as a homogenizing factor in the hierarchical organization of nature. To Galápagos, the conservation of animal mutualists should have a strong positive side effect upon the preservation of large parts of the native flora, which, however, risk extinction because the same animals are reused *en masse* as double mutualists and because of their predator naivety often fall as the first victims to human colonization of oceanic islands^41^.

## Materials and Methods

### Study areas, periods and sampling

The Galápagos Islands lie on the Equator in the Eastern Pacific Ocean, 960 km off the coast of South America. This 0.5–4 MY old volcanic archipelago is composed of 18 islands larger than 1 km^2^ and numerous smaller islets.

Galápagos has an extant flora and fauna of ~1,400 species of flowering plants (~60% of these are introduced), ~2000 species of insects (~45% introduced), 35 species of land birds (9% introduced), and 26 species of lizards (19% introduced), and finally, the giant tortoises^54–56^.

We made short expeditions to 12 of the islands in 2010–2014. In 2010.02.07–22 (year.month.from day-to day), we visited the islands of Floreana, Fernandina, Pinta, and Santiago; in 2011.02.04–26, Fernandina, Pinta, Santiago, San Cristóbal, Santa Cruz, and Santa Fe; in 2012.02.04–18, Española, Santa Fe, Genovesa, and Marchena; in 2013.02.18–28, Santa Cruz, Isabela, Pinzón, and Floreana; and in 2014.04.26–05.15, San Cristóbal, Fernandina, Isabela, Pinta, and Santiago). Six of us (AT, BR, JMO, MN, PV, RHH) spent single days on Floreana, Santa Fe, and Pinzón, and periods of three consecutive days on the other islands.

In addition, during an uninterrupted stay by one of us, RHH, from March 2010 to February 2011, data were collected from a set of sites on the two most human-populated islands, Santa Cruz and San Cristóbal: Eight sites on Santa Cruz (in the lowland near Puerto Ayora and in the highland at Media Luna) and San Cristóbal (lowland: La Galapaguera; highland: El Junco, see^4,10^ for locality coordinates) were sampled with equal effort. Each site was visited every other week during the fruiting season (see^10^ for a table of monthly fruit availability in study areas; most species fruited in the first half of the year), if weather conditions allowed, from February to July during the hot and rainy season (64–2769 mm at coast level), and once per month during the drier and colder season (almost no rain in the lowland). During the study years, January-May 2010 was an El Niño episode, *i*.*e*. very hot and humid, according to the ONI index (Oceanic Niño Index)). On these two islands, we ran 18 mist net sessions. Sampling effort was estimated to be similar on Santa Cruz and San Cristóbal: ~1180 net meter hours/(month x island). Mist nets were opened at sunrise and stayed open for six hours, and were inspected every 20 min for birds. The length of each net was 9 m. In 2011, the sites were resampled but only during the flowering peak (hot season) between January and May.

In addition, mist nets were run on ten other islands (Floreana, Fernandina, Pinta, Santiago, Santa Fe, Española, Genovesa, Marchena, Isabela, and Pinzón), but only in the lowland, three days and five people per island: ~486 net meter hours/(month × island)^4^.

A sampling unit was an individual bird and each unit informed us about interactions between a bird individual and the flowering and fruiting plant species it had visited. At each study site, a total of 2,463 birds were marked with individually numbered metal rings and only one pollen load sample and faecal dropping were collected per individual in order to avoid pseudo-replication (Supplementary Table S4). However, if the same bird was captured on different months, the captures were considered as independent sample units. Although recaptures of marked birds were relatively rare (8% of ringed birds, Supplementary Table S4).

Upon arrival at an island, we first explored an area of about 1 km^2^ around the disembarkation point (mainly for logistical reasons, because we were not allowed to overnight on islands), and recorded all plants in flower for our pollen reference collection. Plant identifications followed ref.^57^ and information available at the Charles Darwin Foundation Herbarium. Nine hundred and ninety pollen smears were made from beak, throat and forehead of individually sampled birds by swabbing a small cube (3 mm^3^) of glycerine jelly, stained with fuchsine, on their beak and peri-mandibular feathers. The gelatine cube was then placed on a microscope slide, which was covered and melted by a weak heat source (a lighter) to produce a single layer of stained pollen grains. Preparations were sealed with clear nail polish, labelled and stored. Pollen grains were later identified by means of a reference collection and counted under a light contrast microscope at the Charles Darwin Station^58^. Data from each site were pooled and used to build year-round pollination networks. Interaction strength in the constructed networks was estimated as mean number of pollen grains/mm^2^. All pollen grains within an area of 79–504 mm^2^ on the slide were counted, varying with pollen grain density (Supplementary Table S4).

Captured birds were left for up to 30 min in ringing bags to defecate. Their droppings were individually stored to evaluate seed dispersal. Intact seeds in 2,348 droppings were extracted and identified under a dissecting microscope by comparison with a seed reference collection in the Charles Darwin Foundation^59^. Viability of seeds was tested in two ways: (1) seeds were sown in trays of 104 units filled with a substrate composed of agricultural soil, volcanic lapilli and turf (2:1:1 ratio). In a shaded greenhouse, soil in trays was kept moist throughout the entire experiment. Seedling emergence was recorded for two years: every other day during the first year and once a week during the second year. In addition, (2) we performed a seed viability analysis, by applying 2,3,5 triphenyl-2H-tetrazolium chloride diluted to 0.1% for 24 hr in the dark and at room temperature. Data from each site were pooled and used to build year-round seed dispersal networks. Interaction strength in the constructed networks was estimated as the mean number of viable seeds of a plant species per dropping per bird species (Supplementary Table S4).

All permissions to do field work on all islands were granted to us by the Galápagos National Park Directorate, which also approved all our experimental protocols and the study in general. In addition, all methods were performed in accordance with the relevant guidelines and regulations from the Directorate. All our applications and permissions are archived at the Galápagos National Park Directorate, Puerto Ayora, Santa Cruz, Galápagos.

### Analysis

Hitherto, 1-layered ecological networks, *i*.*e*. isolated networks of only one kind of interaction, are by far the most studied. However, recently, network analysis has adopted a multi-layered approach^60^, in which variability in time, space and link type is stressed. As we understand the definitions of different multi-layered networks given in Ref.^60^, our study network is type *c* in their Fig. 1 (“Networks with different interaction types that are connected through shared species. Layers are ‘diagonally coupled’, so interlayer edges occur only between shared species”^60^). Here, we pooled pollination and seed dispersal data from all study sites in order to build an all year-round qualitative, 2-layered *plant-bird mutualism network*. In the future, most network metrics have to be reassessed when the field moves from 1- to multi-layered network analysis, *e*.*g*. should the potential number of links be 2 x no. animal species x no. plant species, when we calculate connectance in a 2-layered network. Together with the *plant-insect pollination network* our study network constituted the *plant-insect/bird mutualism network* of the archipelago (Fig. 5). Such a combined network is here termed a super-network^52^. ‘Qualitative’ means that only information about presence or absence of interactions between species was used. We did obtain link strength data expressed as pollen load size in the pollination network, and as number of viable seeds per dropping in the dispersal network. However, this information was only used in a comparative analysis between single and double mutualists and mutualisms of species dependencies and strength^61^, keeping the two kinds of interactions (pollination, seed dispersal) separated. We did that because we were unable to find an adequate exchange rate between the two kinds of link strength measures, which would be needed in order to build a robust, quantitative, multi-layered plant-bird mutualism network (but, see^62^ for an example). However, we stress that no two links are alike in the real world, not even within classic 1-layered networks like a pollination network.

We analysed the level of network nestedness *NODF*, *i*.*e*. the extent to which the link set of a species is nested within the link sets of other species of its community^19^. In order to do so, we used the *ANINHADO* algorithm, which calculates the *NODF* value for a given network. The value of the *NODF* parameter lies within the interval [0 (non-nested) and 100 (perfectly nested)]. The significance of the empirical *NODF* value was estimated by comparing it to a distribution of *NODF* values generated from 1000 random networks of a size similar to the empirical one and constructed according to a specific null model termed *Ce* (see^63^ for details). We used information about the nested structure of the network to locate the position of the cluster of double mutualists in the plant-bird mutualism network.

In the plant-bird mutualism network, we also looked for small groups of highly linked species or modules^20,64^. To do so, we made a modularity analysis, using the software *NETCARTO* ^65^, which calculates level of modularity *M* and the number of modules, and describes the identity and distinctness of each module in the network. The significance of the empirical *M* value was estimated by comparing it to a distribution of *M* values generated from 100 random networks of a size similar to the empirical one and constructed according to a given null model (see^20,65^ for details).

In order to quantify network centrality of each species and the extent to which species connect the pollination and seed dispersal parts of the network, we calculated the betweenness centrality of each species of bird and plant, using the software *PAJEK* ^66^. Betweenness is a scaled measure of the centrality of a species *i*, based on the number of shortest paths from all species to all others, that pass through *i*.

## Electronic supplementary material

Supplementary Figures S1–2

Supplementary Tables S1–5

Supplementary Datasets S1-3
