# Supplementary material for: Disclosing the double mutualist role of birds on Galápagos
Source: Sci Rep. 2018 Jan 8;8:57. doi: 10.1038/s41598-017-17592-8 (PMC5758524; doi:10.1038/s41598-017-17592-8)
Supplement: Supplementary file 1 — Supplementary Figures S1–2 [file 41598_2017_17592_MOESM1_ESM.pdf]

J. M. Olesen, C. F. Damgaard, F. Fuster, R. H. Heleno, M. Nogales, B. Rumeu, K. Trøjelsgaard, P. Vargas & A. Traveset. Disclosing the double mutualist role of birds on Galápagos.

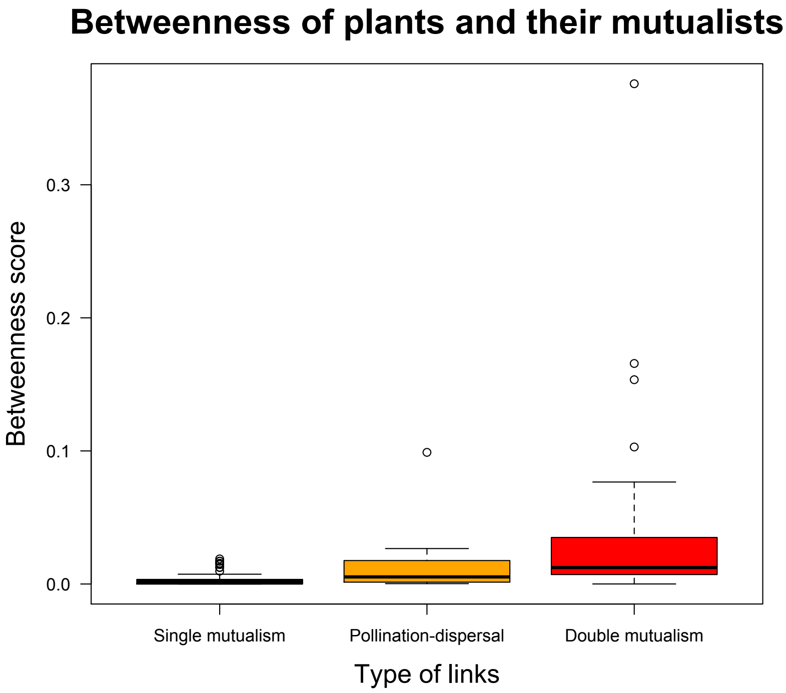

Supplementary Figure S1. Variation in betweenness in the Galápagos plant-bird network. Betweenness is a scaled measure of the centrality of a species *i,* giving the number of shortest paths from all species to all others, that pass through *i*. 'Single mutualism' (black) is either pollination or seed dispersal; 'Pollination-dispersal' (orange) is interactions between species involved in both kinds of mutualisms but not to the same species; and 'Double mutualism' (red) is interactions between species, which are involved in at least one double mutualism. The box-and-whisker plot is based on the coloured second quartile (*IQR*), and the two whiskers represent the first and the third quartiles, *Q1* and *Q3*, resp. Outliers (unfilled circles) have a betweenness score > *Q3* + 1.5 *IQR*.

J. M. Olesen, C. F. Damgaard, F. Fuster, R. H. Heleno, M. Nogales, B. Rumeu, K. Trøjelsgaard, P. Vargas & A. Traveset. Disclosing the double mutualist role of birds on Galápagos.

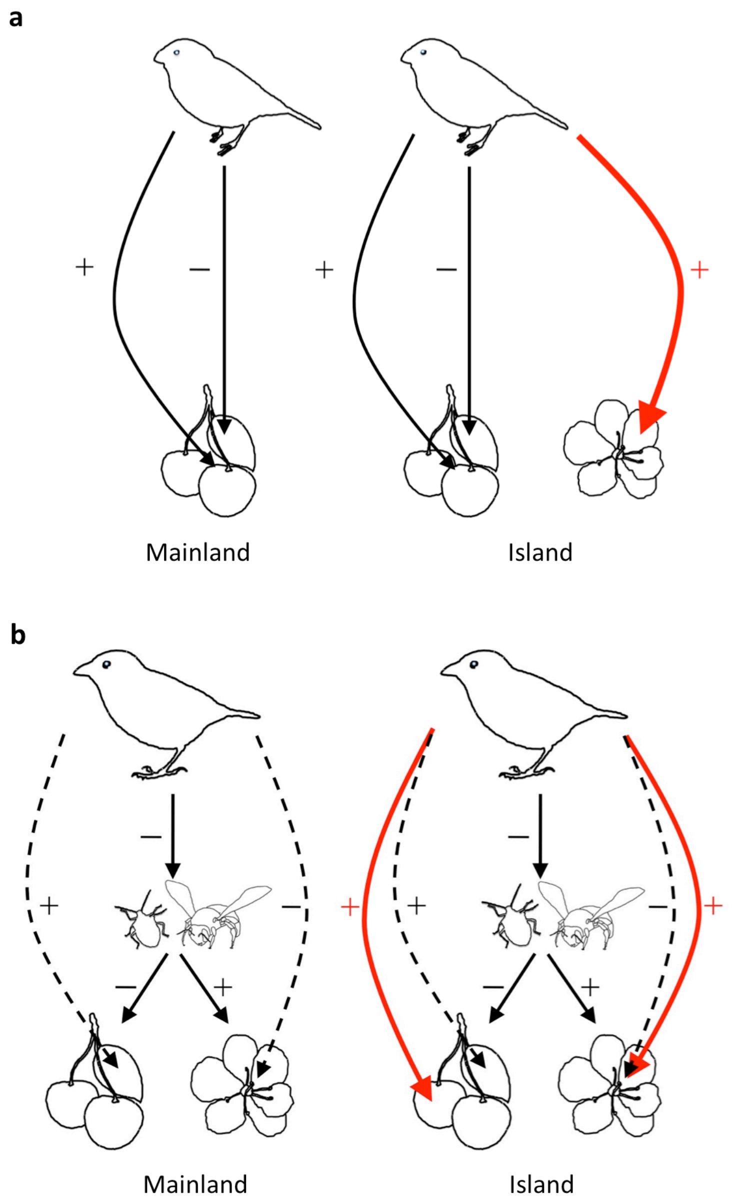

Supplementary Figure S2. Mutualistic (+) and antagonistic (–) interactions, interaction release and cascade in a bird-insect-plant motif. **a**, frugivore/seed disperser also pollinating flowers on islands; **b**, arthropod-eater also dispersing seeds and pollinating flowers on islands. Solid and dashed lines are direct and indirect effects, respectively. Red-coloured interactions are the specific island interactions.
